# RURAL SYSTEM-BASED COPING STRATEGIES FOR ADVANCING RESEARCH AND HEALTHCARE WHILE TRANSITIONING TO THE NEW NORMAL

**DOI:** 10.1093/geroni/igac059.369

**Published:** 2022-12-20

**Authors:** Joan Ilardo, Angela Zell

## Abstract

The pandemic brought about many changes at a system level that were necessary to facilitate continuity of research and the practice of healthcare. Rural and underserved populations were and continue to be the hardest to reach both before and during the COVID-19 pandemic. Therefore, the innovative coping strategies that were implemented during the pandemic are essential to continue as we transition into the endemic stage and realize our new normal as researchers and practitioners. This symposium will highlight adaptations made to research and practice during the COVID-19 pandemic that will apply to future system-level strategies. Drs. Wiese and Park will present results from an online exercise intervention for rural older adults that are at risk of cognitive decline. Drs. Ilardo and Zell will present a roadmap inspired by the pandemic to promote food equity. Drs. Zell and Haque will address rural solutions to polypharmacy issues using an interdisciplinary team approach. Dr. Monaghan-Geernaert will discuss important implications that international caregivers have in fulfilling caregiving gaps in the US, followed by Dr. Freeman et al.’s discussion of rural, long-term care facilities (LTCF) experiences during the pandemic. These system-based coping strategies have policy implications for rural healthcare as we transition to the new normal.

